# Challenges and opportunities for implementation and dissemination of a task- sharing counselling intervention for depression at primary health care level in South Africa

**DOI:** 10.1186/s13033-023-00575-w

**Published:** 2023-03-30

**Authors:** One Selohilwe, Lara Fairall, Arvin Bhana, Tasneem Kathree, Babalwa Zani, Naomi Folb, Crick Lund, Graham Thornicroft, Inge Petersen

**Affiliations:** 1 Centre for Rural Health, School of Nursing and Public Health, University of KwaZulu-Natal, Durban, 4001 South Africa; 2 Knowledge Translation Unit, University of Cape Town, Cape Town, South Africa; 3 School of Life Course & Population Sciences, King’s College London, London, UK; 4 Health Systems Research Unit, South African Medical Research Centre, Durban, 4091 South Africa; 5 Alan J Flisher Centre for Public Mental Health, Department of Psychiatry and Mental Health, University of Cape Town, Cape Town, South Africa; 6 Centre for Global Mental Health, Health Service and Population Research Department, Institute of Psychiatry, Psychology and Neuroscience, King’s College London, London, UK; 7 Centre for Global Mental Health and Centre for Implementation Science, Institute of Psychiatry, Psychology and Neuroscience, King’s College London, London, UK

**Keywords:** Task-sharing, Implementation, Dissemination, Lay-counselling, Depression, Psychosocial intervention

## Abstract

**Background:**

The treatment gap for mental health services is a growing public health concern. A lay-counselling service located at primary health care (PHC) level could potentially help to close the large treatment gap for common mental disorders in South Africa. The aim of this study was to understand multilevel factors contributing to implementation and potential dissemination of such a service for depression at PHC level.

**Methods:**

Process qualitative data of the lay-counselling service for patients with depressive symptoms was collected alongside a pragmatic randomized controlled trial evaluating a collaborative care model that included a lay-counselling service for patients with depressive symptoms. Semi-structured key informant interviews (SSI) were conducted with a purposive sample of PHC providers (lay-counsellors, nurse practitioners, operational managers), lay-counsellor supervisors, district and provincial managers, and patients in receipt of services. A total of 86 interviews were conducted. The Consolidated Framework for Implementation Research (CFIR) was used to guide data collection as well as Framework Analysis to determine barriers and facilitators for implementation and dissemination of the lay-counselling service.

**Results:**

Facilitators identified include supervision and support available for counsellors; person focused counselling approach; organizational integration of the counsellor within facilities. Barriers included lack of organizational support of the counselling service, including lack of counselling dedicated space; high counsellor turnover, resulting in a counsellor not available all the time; lack of an identified cadre to deliver the intervention in the system; and treatment of mental health conditions including counselling not included within mental health indicators.

**Conclusions:**

Several system level issues need to be addressed to promote integration and dissemination of lay-counselling services within PHC facilities in South Africa. Key system requirements are facility organizational readiness for improvement of integration of lay-counselling services; formal recognition of counselling services provided by lay counsellors as well as inclusion of lay counselling as a treatment modality within mental health treatment data element definitions and the need for diversification of the roles of psychologists to include training and supervision of lay counsellors was also emphasized.

## Introduction

A large proportion of people living with mental disorders do not receive the treatment they need and the majority of these people are found in low- and middle-income countries (LMICs) [1]. Research shows between 76 and 84% of people who need services for serious mental disorders in LMICs do not receive care and treatment [2]. Further, globally, of those receiving treatment only 41% receive treatment that meet minimal standards [3]. The depression treatment gap is of particular growing public health concern as it is the leading cause of disability worldwide [4]. Further, untreated depression comorbid with HIV and non-communicable diseases (NCDs) compromises the fight against the HIV epidemic and the rising burden of NCDs [5–7].

In South Africa (SA), there is similarly a large treatment gap, particularly for common mental disorders (CMDs) [8], with depression being the most prevalent individual mental disorder [9]. With a high prevalence of depression in people with chronic conditions [7] leading to worse health outcomes [10–12], there is an urgent need to increase access to mental health treatment services for patients with chronic conditions. In terms of policy imperatives, South Africa ‘s National Mental Health Policy Framework and Action Plan (2013–2020) [13] commits to providing equitable and decentralized integrated primary mental health services that includes services for people with chronic conditions. Further, the Integrated Clinical Services Model (ICSM) provides an enabling health systems strengthening approach for the integration of mental health services. It has a patient-centred focus aimed at achieving operational efficiency at facility level, improved patient clinical outcomes and an informed population who take individual responsibility for their health [14]. However, implementation of these reforms remains a challenge, with implementation and dissemination research required to assist with implementation and scaling up of evidence-based implementation packages into routine practice for integrated primary mental health care [15–17].

The PRogramme for Improving Mental health carE [18] in South Africa (PRIME-SA) developed a collaborative care model for depression, alcohol use disorders (AUDs) and schizophrenia in primary health care settings [19] to produce evidence on the implementation and scaling up of integrated packages of care for depression and other priority mental disorders. A key innovation within the collaborative care model for depression was the introduction of a facility-based depression lay-counselling service. In this model, nurses identify patients with depression, and refer those with moderate to severe symptoms to doctors for initiation of anti-depressant medication. Patients with mild to moderate symptoms were referred to the lay-counsellors for structured manualized depression counselling under the supervision of a psychologist or registered psychological counsellor [20].

A non-randomly assigned comparison group cohort study indicated that patients referred for care within the collaborative care model had significant clinical improvements in depressive symptoms compared to those who were not referred [21]. A pragmatic cluster randomized control trial where the main outcome was a reduction in depressive symptoms demonstrated non-inferiority of the collaborative care model to usual care where counselling was provided by mental health specialists for hypertensive patients [22]. Being a pragmatic trial, participants were recruited and enrolled into the trial independently of the intervention, so as to optimize real world applicability of the trial results. A limitation of this design was low exposure of the trial participants in the intervention clinics to the task- sharing facility-based counselling service within the collaborative care model. This paper reports on the process evaluation undertaken in parallel with the trial to understand multilevel factors contributing to this low exposure, as well as potential enabling factors that need to be taken into account in future efforts to implement and scale up tasked-shared psychosocial counselling for depression in South Africa.

## Methods

### Counselling intervention description

The counselling intervention was a structured, manualised eight session brief counselling intervention informed by adapted cognitive behavioural interventions, including problem solving, cognitive restructuring and behavioural activation [23]. An adherence to chronic medication session was added to total nine sessions. Session one focuses on depression psychoeducation and session eight on closure of the intervention. Sessions two to seven focus on addressing triggers and causal influences of depression previously identified through formative work [24], namely poverty; interpersonal conflict; social isolation; grief and bereavement; and internalised and externalised stigma. Each of these triggers are addressed using a specific cognitive behavioural intervention [20].

### Referral process

The lay-counsellors provided waiting room educational talks on depression every morning to patients waiting to be seen by a PHC nurse. The counselling intervention was nested within a depression collaborative care model. Within this model, nurses identified patients with depressive symptoms using Adult Primary Care (APC) clinical decision support tool, also known as the Practical Approach to Care Kit [25]. Nurses were trained in strengthened mental health guidelines of APC whereby patients identified with moderate to severe depressive symptoms were referred to the doctor for anti-depressant initiation [23]. Patients with mild and moderate depressive symptoms were referred to the lay-counsellor-led psychosocial counselling intervention [19]. Upon referral to the counselling services, the patient was initiated into the intervention and taken through the first session of psychoeducation on the same day. The patient’s most pressing need was collaboratively identified with the lay-counsellor during the first session and facilitated during the next follow up session. A regular day and time for follow-up sessions were also agreed upon during the first session and a patient appointment card provided for a visual reminder. The second session was followed by an adherence session for patients on chronic medication before going through the rest of the sessions. The sessions were 45 min to an hour each and the lay-counsellor provided telephonic reminders of the appointment before each session.

### Training and supervision of lay-counsellors

Project- employed lay-counsellors received regular supervision and mentoring for continuous support and development. Following training, they each received onsite in vivo supervision through the apprenticeship model for all eight sessions until competency was achieved. Counsellors replacing those who had resigned received structured peer to peer learning where they were placed with a more experienced counsellor before they were placed in their own facilities. Further, all the lay-counsellors received regular offsite weekly group supervision, and they could contact the supervisor telephonically whenever they needed to. Supervision was provided by registered psychological counsellors and intern clinical psychologists [23].

### Study site

The study was nested within the PRIME-SA trial study site in the Dr Kenneth Kaunda (Dr KK) district in the North West province of South Africa [26]. The district had 31 primary health facilities and 10 community health centres [27] servicing a population of approximately 796 823 [19]. The clinics are staffed by nurses and rotational medical officers with in-reach services provided by two district psychologists, with a referral outpatient psychology clinic available at the district hospital.

### Study design

A descriptive qualitative research design was adopted to systematically examine stakeholder perceptions of key barriers and facilitators to implementation of the counselling intervention. This qualitative data of the lay-counselling service for patients with depressive symptoms was collected alongside the pragmatic randomized controlled trial previously described [22, 26]. The Consolidated Framework for Implementation Science (CFIR) informed both the data collection and analysis. CFIR provides a comprehensive organising framework for systematically identifying factors at multiple levels that influence implementation [28, 29]. It comprises 39 constructs divided into 5 domains namely outer setting (four constructs); inner setting (14 constructs); intervention characteristics (8 constructs); characteristics of individuals (5 constructs) and process (8 constructs) [29] (appendix 1). Using the CFIR framework, the outer setting for the study reflects the broader South African health policy landscape at a national and provincial level as well as the socio-economic and political context of Dr KK district that influences patient needs and resources. The outer setting is mediated by the inner setting, made up of the primary care facilities and district authorities. The individual domain comprises facility managers, nurses who provide consultation services in primary health facilities, lay-counsellors who provided the counselling intervention, and service users in receipt of the intervention. The intervention domain was focused on understanding enabling and challenging aspects of the lay-counselling intervention for depression.

## Recruitment and sample description

### District and provincial managers

Purposive sampling was used to recruit provincial managers responsible for coordinating and planning of primary health care services and managers at district levels managing primary health services. They were contacted and provided with a brief explanation of the study (indicated on Table 1) and request for an interview. Those who consented were then given an interview appointment date.

**Table 1:** Participants interviewed per category

Participants	N	Interview focus
Provincial manager	1	The impact of integrated mental health services; systemic barriers to implementation and how these could be overcome, as well as planning and budgetary allocations for mental health
District Managers	7	
Facility Operational Managers	7	The operation of their facilities including their leadership skills; staff training; task- sharing; having the lay-counsellor in their facility; human resources and information systems
Professional Nurses	14	The factors related to their role in the intervention in identification and referral of patients with depressive symptoms, as well as case managers
Project- employed Lay-Counsellors	8	Perceived reception of the counselling service and the counsellors’ experiences of the service and service integration into the facility
Counsellors in the system (HCT Lay-Counsellors)	19 (4 focus groups)	Their perspectives on the possible expansion of their role to include providing counselling for patients with depressive symptoms
Patients	28 (7 focus groups)	Their experience of the lay-counselling intervention
Counsellor Supervisors	2	Counselling intervention supervision experience
Total	86	

### Service providers

Professional nurses including facility operational managers, and lay counsellors were purposively sampled through organisational structures and contacted with a request for an interview and an appointment following a brief explanation of the study. Individual nurse counselling referral rates from each of the intervention facilities in the trial were identified from the intervention process data. High and low referring nurses were differentiated using this data and approached to participate in the study. Lay counsellors included both project-employed counsellors as well as existing HIV Counselling and Testing (HCT) counsellors as potential providers of the counselling service. Registered psychological counsellors who provided supervision were also included in the study (See Table 1).

### Clients/ patients/ users

Patients who had received at least one counselling session were identified through intervention process data and contacted (See Table 1). Those willing were grouped according to the number of counselling sessions received; 1 session; 2 sessions and 3 or more sessions. The patient participant groups were provided with an appointment date to meet with the interviewer at their primary health facility where focus group interviews took place in a private room (See Table 1).

The study was explained to the participants on the day of the interview before they provided written informed consent to participate in the study. The participants were provided with a copy of the consent form for their records. Illiterate participants had the consent form read out to them verbatim in the presence of a witness who countersigned when they provided consent by marking a cross (x). Participation was voluntary and participants were informed they could withdraw from the study any time they chose to.

### Data collection

Qualitative data was collected using individual audio recorded semi-structured interviews guided by CFIR constructs with stakeholders listed in Table 1. Individual interviews and focus groups were conducted with the counsellors and patients (as indicated in Table 1). In addition to interviews, data were collected from observational field notes of the counselling intervention; and minutes of meetings with district health department personnel. All interviews were carried out by members of the research team who were bilingual Setswana /English speaking registered psychological counsellors and psychologists trained in conducting individual and focus group qualitative interviews.

Interviews focused on barriers and enablers of implementation of the task-shared counselling intervention along the CFIR dimensions that were deemed applicable for the different groups of participants (see Table 1).

### Analysis

Framework analysis using NVivo framework matrix (version 11) was used to analyse and interpret the data collected from qualitative interviews. Framework analysis allows for predetermined themes while leaving space for discovering new ones [30, 31]. The authors followed seven stages informed by Ritchie and Spencer’s framework analysis method [30]. Interviews were first transcribed verbatim and where interviews were conducted in local languages, were translated into English, with back translation checks applied. In step two the researcher immersed herself in the data by reading and re-reading the transcripts. Initial coding was carried out by two researchers, using the CFIR domains and barriers and facilitators as pre-determined themes and emergent themes expressed by participants. This was an iterative process with the framework continually getting refined as well as clarification of how each theme should be used. The initial coding was used in step four to develop the analytic framework by the researcher. Step five involved coding the transcripts using the analytical framework. The data were then summarised and charted into the framework matrix in step six prior to interpretation (step seven). The different stakeholder groups were analysed separately under the different CFIR domains and constructs before identifying cross cutting themes across the stakeholders. Themes were discussed with co-investigators and finalized after consensus.

### Ethical considerations

Ethical approval for the study was obtained from the University of KwaZulu-Natal’s Biomedical Research Ethics Committee (BREC) (reference BFC049/15) Permission to conduct the study was granted by the North West Provincial Department of Health.

## Results

### Intervention domain

#### Facilitators

The professional nurses (PNs) interviewed indicated that the APC guidelines were helpful in identifying and referring patients, with twelve out of fourteen (85.7%) nurses interviewed indicating that they used APC when identifying and referring patients with depressive symptoms.

We have to give the best patient care so by following the guideline you basically doing that

(PN9)

A total of six out of eight (75%) project-employed lay-counsellors reported that it was helpful to have referred patients linked to them by being called to the consultation room for introductions or the nurse walking the patient to the counselling room. Patients would often be lost to the counselling intervention if this did not happen.

The only challenge I had was … when sister is referring the patients, they don’t bring the patients to me, they just tell the patients go to [the counsellor] and the patients are hungry and tired by then. They just go home once they are done with the nurses.

(Counsellor9)

The intervention was reportedly acceptable to the patients in all the focus groups. They reported improved depressive symptoms including improved sleep, decreased irritability, reduced worry, improved mood, and no longer having suicidal ideation.

It has also helped me a lot because I was someone that struggled to sleep or I would cry in my sleep […] Now I sleep well.

(patient, focus group4)

If you have not received counselling some of these challenges will play with your head. Counselling has taught me what to do when I am going through certain challenges and it has also opened up my mind.

(patient, focus group6)

I have now come to see counselling is important because I now feel like human being […] I couldn’t believe that one day I would be able to let things go and still live.

(patient, focus group3)

Patients specifically identified problem-solving skills as helpful for tackling life problems, including practical problems like not having an income/job as well as relationship problems.

[counselling] has helped me to look at other ways I can bring in an income instead of only looking at the money I receive from work. There is something that I am doing on the side to bring extra income.

(patient, focus group4)

I have gone back to school after I dropped out following completion of my N6.

(patient focus group4)

Receiving feedback on the progress of patients referred for counselling was identified by professional nurses as an incentive to identify and refer patients with depressive symptoms. A total of ten (71.5%) professional nurses reported receiving feedback on patients they had referred for depression counselling. Reassessment of patients who had completed their treatment was reported by 57% (eight out of 14 professional nurses). PN7 talked about how reassessing a patient on completion of the intervention was rewarding for her.

I have [re-assessed a patient], and they told me that the problems they had were resolved […] I felt happy that at I had helped someone.

(PN7)

#### Barriers

A few nurses, four out of sixteen (21%), all of whom had low referral rates, discussed difficulties in differentiating between depressive symptoms and socioeconomic problems which trigger depressive symptoms, potentially affecting referrals made to the lay-counsellors.

[…] sometimes you get a patient who is trapped in poverty and you don’t know if they are depressed or not. You don’t know how to categorise it as these things are not even on the guideline.

(PN1)

### Inner setting

#### Facilitators

##### Counsellor organizationally integrated into facility

Only half of the project-employed counsellors (four out of eight) reported they felt accepted organizationally as part of the facility staff (i.e. provided feedback regarding patients, were considered as staff members and included in staff meetings). Being organizationally integrated reportedly helped build their confidence, validate their role and encourage referrals from nurses for patients identified with depressive symptoms.

I was part of the team and I felt that the job I do is important to the team.

(Counsellor2)

I usually attended staff meetings and sometimes they would want to know about my work and how I am coping. It was then where I would communicate about patients and work. [This was helpful] because they would also motivate each other to start referring.

(Counsellor5)

##### Supervision and support for project-employed counsellors

All lay-counsellors reported supervision was helpful for developing their confidence and providing emotional support. Group supervision provided a community of practice learning space that was appreciated by all counsellors. Peer to peer supervision was reported to be especially helpful with skills development. Areas identified for improvement with respect to their supervision included having one-on-one onsite supervision and emotional support more regularly.

I was happy because I felt that I was taking the patient’s problem as my own, so he (the supervisor) helped me not do that….

(Counsellor2)

[Supervision could be improved] by making it a regular thing, because sometimes they would come and then take a while to come back again, and the supervision onsite is better because the focus is solely on you, unlike the [group] supervision.

(Counsellor5)

#### Barriers

##### Counsellor and counselling activities not integrated into the facility

Half (n = 4) the project- employed counsellors reported not feeling integrated into the facility and facility activities. This was reflected in not being given adequate space to work, not being included in staff meetings and generally feeling marginalized, with the counselling service not recognised as important. This had a negative impact on counsellors’ confidence and the programme generally.

[…] I didn’t have a good relationship with the facility manager […] she was not the kind of person who would want to know how is it going with my work, if I had space to work in and if I was fine. I had to stand up for myself to secure space to work in

(Counsellor4)

[…] at first, they did not refer and I had to prove myself in order to have a working relationship with the sister. They didn’t see me as part of the facility- “no you are for depression and you are for PRIME”. It was difficult but I managed to pull through

(Counsellor8)

##### Lack of supervision and support for nurses

Lack of supervision and support for the nurses was another barrier. Only three out of fourteen nurses (21.4%) (all who had high referrals) reported having received APC supervision and support. The nurse below discusses how she had to make her own way through with the APC guidelines when assessing patients.

Most of the time I am on my own when using this [guide], I don’t really have support

(PN4)

##### Space

Lack of dedicated counselling space was discussed as one of the main barriers in the inner setting by all of the eight lay-counsellors interviewed. The counsellors below discuss how the space challenge frustrated them and compromised patient privacy.

I have been working outside without a counselling room for a long time. I think that is one of the reasons patients don’t come back especially in winter because we have our sessions outside due to lack of space.

(Counsellor2)

[lack of space] affected my work a lot, because I realised the patients were losing their trust in me as they observed I don’t have a dedicated space to work in

(Counsellor4)

[..] You struggle to get a place to work in, and that is frustrating even for patients because they never know where to find you

(Counsellor5)

The need for space was also reported as an intervention challenge by patients. Group members from four focus groups reflected on how space shortage at their facility frustrated the intervention.

We moved from one place to another. I never knew where to go when meeting with her for follow up sessions. I would ask myself if she will be in the room she used previously.

(patient; focus group 6)

[the counsellor] Had a serious challenge with space. If she couldn’t secure a space for this week, she would make an effort to call you the following week and ask if you could come in to make-up for the missed session.

(patient; focus group 8)

##### Lack of a consistent facility-based counsellor

A total of six of the project-employed lay-counsellors (60%) from the ten facilities resigned during the course of the study. This resulted in a total of six facilities (60%) having a part-time facility-based counsellor or no counsellor at all for certain periods during the study. Patients from three focus groups discussed how the high turnover meant they could not always get counselling services at their facilities.

[…] I came for the 3rd session and I was told the counsellor was no longer available and that [the facility was] still busy trying to organise another counsellor so I’ve been sitting at home ever since waiting

(patient; focus group5)

##### Nurse emotional needs not addressed

All the professional nurses interviewed identified the need to receive help for their own emotional problems, which was accentuated by emotional labour demands of dealing with patients’ mental health problems. This was corroborated by 71% (five out of seven) of the facility managers interviewed. Nurse PN1 and facility manager FM8 discuss their experience of dealing with patients’ mental health problems.

[…] I also have my problems, and I need to be debriefed so that I can be able to help my patients. It becomes difficult to help someone who is depressed when you also have a problem

[..] patients sometimes tell me painful stories which bring a heaviness and cause me to lose control of the situation. There are times where I felt like crying

(PN1)

It’s easier to listen to the patients when they are talking about the physical part of their problem. The problem is when they go deeper with sensitive issues and we put ourselves in their shoes especially if you are experiencing something similar [..] You end up not giving enough or you don’t dig enough and you are left with a burden as well …

(FM8)

District managers (DMs) also identified a need for a functional employee assistance programme (EAP) to help nurses and other staff members with their emotional needs.

Yoh, that one is a big gap for us [..] Our psychologist sees patients as well as assisting us with our EAP programme, although not structured.

(DM6)

There are programs but the efficiency and effectiveness of our [EAP] programs is a challenge. […] I will say that it is an area where we need to improve.

(DM5)

Not having a dedicated mental health coordinator at district level was reported as a barrier to scaling up the lay-counselling intervention. All district managers reported shortage or a complete lack of mental health coordinators.

There is no specific person […] coordinating [mental health integration] at the district [level] there is none. Only one sub district has a coordinator [..] This means it is not taken seriously when there is supposed to be somebody at the district overseeing the integration.

(DM4)

### Individual characteristics

#### Facilitators

##### Nurse characteristics

Nearly 60% (eight out of fourteen) professional nurses reported confidence in their ability to identify depressive symptoms following APC training with the majority (5 out of eight) in the high referring stratum.

The need to understand how providers are coping emotionally with their own issues emerged as important with not all providers being able to engage in emotional labour all the time.

We have different personalities. We have a sister who is stronger in the emotional aspect and I give her the patient if I feel like I can’t take anymore. She takes over

(FM8)

##### Counsellor characteristics

Common counsellor qualities reported by patients to be helpful in all the focus groups included the counsellors being professional, empathic, capable, understanding, patient, knowledgeable about the intervention, observing confidentiality, having warm-regard towards the patients, and creating spaces where patients felt safe to share their stories.

She was well trained in our first encounter because she fully explained everything and she was clear. We understood each other that is why I say she understands her job well.

(Patient; focus group 5)

Before reading from the book she spoke to me first and switched off her phone and I could see I was going to get the help I needed. I think she is capable.

(Patient; focus group 5)

What made me go back was that I was relieved; I felt like she understood me and cleared my mind. This enabled me to see a way forward so I wanted to continue talking to her.

(Patient; focus group 4)

#### Barriers

##### Counsellor characteristics

Lay-counsellor supervisors (CS) interviewed reported that not everyone is suited to provide counselling services. Emotional intelligence, emotional resilience and being receptive to supervision were some of the qualities perceived to be necessary.

[…] I do not think anyone can be trained to provide psychosocial counselling. I think it needs someone who is aware of their own mental health and someone […] who can recognise when they get overwhelmed and be able to contain themselves.

(CS1)

### Outer setting barriers and suggested resolutions

#### Barriers

##### Budget constraints

Of the district managers interviewed, four out of seven identified the need for a dedicated mental health budget to help with scaling up integrated mental health care, including plans for scaling up of the psychosocial counselling service for depression. In particular, they were frustrated by a lack of budget needed to employ a mental health specialist team which would be responsible for, among other things, providing supervision and support for PHC nurses and facility-based lay counsellors.

[Lack of a dedicated budget] will affect [scale-up] negatively. You cannot do much without a budget.

(DM6)

We were hoping that the mental health specialist teams would take [scale up of integrated system mental health services] on their shoulders […] but we have been blocked because of money, […]

[...] the clinics are trying the integrated chronic care which includes mental health but I am not sure that they are doing it as well as they could be because that is where the team would come in to help and train and support the mentor. [...]

(DM2)

Psychologists were identified as suitable candidates to provide both training and supervision for the task-shared lay-counselling service when scaling up. Department of health district stakeholders discussed the need for these activities to be included in intern and community service psychologists’ job descriptions as part of their outreach activities.

I think the Psychologists should ensure [counsellors] continue being trained and support them.

(DM8)

[…] The psychologists are more hospital based. I even requested for these people even the comm serves, […] to be placed at the sub district which will be very helpful. And to also give them exposure not only in terms of the hospital setting but to do some outreach.

(DM6)

##### Staff shortages

Staff shortages in conjunction with high headcounts as well as patients with multiple conditions was reported as a barrier for scaling up the lay-counselling intervention by 71% (five out of seven) of facility managers. Identification of depressive symptoms was identified as requiring longer consultations, a person-centred care approach, as well as emotional labour that was not possible given the time available for nurse patient consultations.

They [nurses] see so many people. I think that maybe the quality of mental health services provided in the integrated system might not be as good. Obviously because they now have to look at the BP treatment and do the bloods for the HIV and by the way you also need your pill for schizophrenia, they are probably not going to do that good mental assessment […] they have to treat a patient with four illnesses and one of these is mental health […]

(DM2)

##### Siloed approach to HIV counselling and testing

While existing HIV counsellors based in primary health facilities who already provided HIV counselling and testing were identified as the ideal cadre to provide the psychosocial counselling at scale by the district and provincial authorities, there were many mitigating factors raised as well. These included HCT counsellors’ negative response to the expansion of their current role without additional remuneration as well as concerns about who in the system would take on their supervision in the absence of project supported supervisors.

[…] We already have problems with the HIV counsellors’ performance, […] their stipend gets paid from the [conditional HIV grant] and their job descriptions are written according to how they are paid and this does not include mental health sessions. I think this could be problematic or it could become problematic if they decide that no this not what they are appointed to do.

(DM7)

There is one challenge, most of our lay-counsellors are HIV funded so you find that they are supported from the conditional grants via a lot of HIV training. […] Maybe that is why this particular person needs to be appointed not from an HIV perspective but as being a counsellor without limiting it to HIV.

(DM8)

HIV counsellors interviewed indicated a willingness to be trained to deliver the counselling intervention. Over half the participants interviewed (57.9%) reported that the situations they encountered in their line of work often exceeded the counselling capacity and capabilities acquired during their training for HCT counselling. Participants in focus group 3 discussed mental health problems as part of HIV/AIDS counselling and an ensuing need to integrate psychosocial counselling as part of the functions of HIV/AIDS counselling.

We should know more [about mental health disorders] because some patients on ART have psych [disorders].

(HIV counsellor focus group3: counsellor)

I just wanted to know about people that have depression, they do come sometimes and [...] usually we find people that have depression also have hypertension and especially diabetes.

(HIV counsellor focus group3: counsellor)

After post-counselling, it’s not every time that the person accepts [their status] you will see on their second visit that they have not yet accepted [their status] and that’s when mental [illness] appears. […] You often hear that mental health problems are caused by [people] receiving their [HIV] status and they start changing, start thinking about a lot of things and depression kicks in. [...] I really think we need to know more so we can identify the signs and symptoms when they come for their follow up sessions

(HIV counsellor focus group3: counsellor)

District manager 10 acknowledged that lay health workers can be trained to provide counselling for positive patient health outcomes.

the lay-counsellors have shown that it can be done. Far more than what we could have thought of. [..] I mean restoring someone’s dignity in life doesn’t have a price on it and these people do it without a qualification.

(DM10)

Low priority afforded to mental health in monitoring and evaluation indicators.

The need for mental health indicators to be taken seriously and included in existing evaluation and monitoring processes with relevant indicators was identified by three district as well as the provincial managers interviewed.

We, including myself; the district management team must monitor it. Probably as part of our DMT meetings, just like we are monitoring your HAST (HIV and AIDS /STI /TB) programmes. We must come up with indicators for us to see that this integration plan is working, […]

(DM1)

## Discussion

**Fig. 1:**
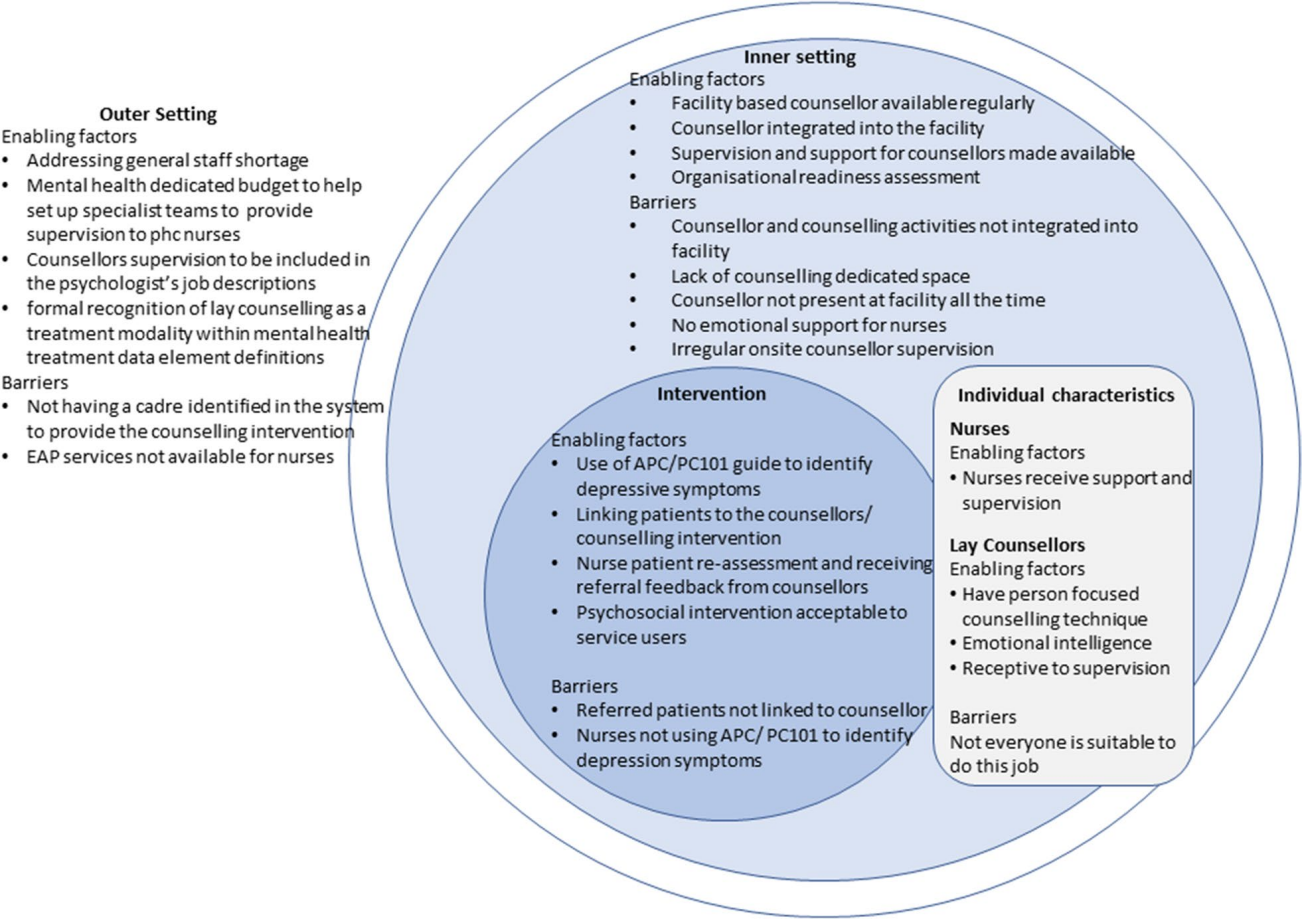
Barriers and facilitators for scale up

This paper sought to explore challenges and opportunities for implementation and dissemination of a task-sharing counselling intervention for depression at primary health care level informed by the CFIR implementation science framework. These are summarised in Fig. 1. In relation to the *intervention domain*, characteristics of the PRIME-SA collaborative care model that helped to promote its implementation and uptake included the APC integrated clinical guidelines. These were reported to be particularly helpful for identifying and referring patients with depressive symptoms. However, this was insufficient to ensure treatment with the practice of nurses directly linking patients identified with depressive symptoms to counsellors reported to be helpful in improving counselling uptake by patients. This finding is supported by a similar finding of a pilot study leading up to the trial [20]. Counsellor feedback on patient progress to nurses as well as re-assessment following completion of the intervention were also reported as promoting greater acceptability of the counselling intervention within the facilities. The importance of teamwork is thus highlighted. Within the PRIME collaborative care model, nurses were identified as the case managers, and carried the responsibility of coordinating care for individual patients across the different health care providers within the model. Receiving feedback on patient progress thus helped to consolidate this case management role of the nurses. With regard to acceptability of the counselling intervention from the patients’ perspective, patient participants reported that the counselling helped to improve their depressive symptoms, with problem solving skills being identified as particularly helpful for addressing socio-economic problems and inter-personal conflicts underlying their depressive symptoms as has been documented elsewhere [6, 32, 33].

In relation to the *inner setting domain* (district and facility settings), organizational integration of the lay counselling service into the facilities emerged as an important enabling factor for implementation of the intervention. However, only half the counsellors interviewed reported being perceived as integral to the functions of the facilities in which they worked. These counsellors reported being included in routine facility meetings where they provided intervention updates and were more likely to have been provided with adequate counselling spaces. Being perceived as part of the collaborative care team communicated the message that both the counselling service and counsellors themselves were valuable to the facility. A previous review of lay counsellors in South Africa, generally indicates high levels of marginalisation of this cadre, with their services not getting due recognition weighing heavily on their sense of self-worth and motivation [34]. This marginalization may have contributed to the high lay-counsellor attrition rate that led to periods when certain facilities did not have a full-time facility-based lay counsellor. High attrition rates of lay health workers is widely documented [34, 35]. The importance of organisational readiness for lay counselling services within PHC facilities to promote implementation and uptake of the service has been previously identified by Sorsdahl et al. [36], whether provided by a designated or dedicated cadre. Supportive facility managers have also been identified as critical in promoting an organisational climate that is supportive of lay counselling services within PHC facilities [37–39]. This includes adequate private counselling spaces found by this study and others [34, 40, 41]. An organisational readiness assessment can help to identify and address potential barriers prior to implementation leading to more effective implementation [37, 38, 42], with some tools having been adapted to assist with this process in South Africa [37, 39, 42]. Lack of counselling dedicated space was also identified as a significant barrier.

Another identified barrier within the inner setting domain was the lack of emotional support for professional nurses tasked with providing integrated mental health services. Existing evidence suggests that high levels of emotional labour increases provider levels of emotional exhaustion, rendering them susceptible to compassion fatigue and burnout [43, 44]. This highlights the importance of emotional support for healthcare workers working with emotionally demanding conditions such as mental health problems. The creation of safe spaces for nurses to process their experiences as well as support from managers and peers has been found to be helpful in dealing with high levels of emotional labour demands in the workplace [43, 44].

Within the *individual domain*, nurses reported that receiving APC training helped promote nurse self-efficacy in relation to diagnosis and referral of patients with depressive symptoms. Self-efficacy plays a role in providing motivation to carry out tasks to achieve set goals such as implementation of a newly acquired skill, with low self-efficacy associated with lack of confidence in ability and/ or lack of preparation and may affect quality of care [45, 46]. Some nurses, however, reported a lack of supervision and support in the use of APC being a challenge for building their self-efficacy in the identification of patients with depressive symptoms; with evidence showing supervision, mentoring and support are important to improve self-efficacy and quality of patient care [46].

In relation to the counsellors, person- focused qualities and emotional resilience were reported as desirable lay-counsellor characteristics by both the patients and lay-counsellor supervisors interviewed; with the importance of selection processes to identify lay counsellors with the necessary person- focused care qualities previously highlighted [20]. Carl Rogers identified empathy, unconditional positive regard and congruence as core features for person- focused counselling [47].

Within the *outer setting domain* (broader policy environment), a number of policy level interventions were identified as necessary to enable integration and optimize scale-up of the facility-based lay-counselling services. The need for budget for a district mental health specialist team, who could, amongst other responsibilities, provide the necessary supervision and support for the lay-counsellors as well as support for the PHC nurses responsible for identification and referral of patients with depressive symptoms was identified. In order to provide supervisors for task- sharing counselling within primary care, the scope of work of intern and community psychologists employed by the DOH was identified as needing to be broadened to include training and supervision of lay counsellors within the system. The need for adequate general staff complements was also stressed so as to relieve the patient load on nurses and allow for more time to adequately assess for depressive symptoms that may not be the immediate presenting problem in patients with chronic conditions.

An intractable bottleneck that has been identified by other studies in relation to lay counselling services in South Africa, is the need to identify a suitable cadre to implement task- sharing counselling within the PHC system [36]. In this study, existing HIV counsellors were identified as a potential cadre by health managers; and HIV counsellors interviewed were supportive of an expansion of their role to include counselling for mental health conditions as they often encountered such conditions in their patients. However, many barriers to an expansion of their role were raised as well. Perhaps one of the most challenging of these was that they were employed via an HIV conditional grant that restricted their scope of counselling to pre and post-test and adherence counselling for HIV patients. Others, perhaps more readily addressed included organisational readiness to integrate them; selection criteria; career advancement and the provision of training for this cadre.

Lastly, in order to support scale-up goals it is essential to expand the mental health indicators in the monitoring and evaluation system along the treatment cascade for mental health in PHC beyond the indicators available at the time of the study that focused on number of patients screened and treated for mental health problems. Ideally the treatment cascade for mental health condition should emulate that for other conditions [48], including screening, assessment, diagnosis, referral for anti-depressant medication initiation if necessary and to the counsellor for counselling; uptake of the treatment and back referral to the consulting nurse for re-assessment. Having indicators at the different steps will help with monitoring and evaluation of integration of the counselling service along this cascade of care [49]. Pence O’Donnell and Gaynes [48] argue patient outcomes can be significantly improved by targeting all the problematic steps along the treatment cascade, as has been the 90–90-90 approach for improving HIV patient outcomes [50]. The inclusion of counselling indicators as part of the ‘number treated’ indicator would also assist with embedding the role of the lay counsellors in the system as service providers. It would also serve to acknowledge the service they provide, thus promoting organizational integration of the lay counselling services within the health system [51].

## Limitations

While efforts were made to ensure an adequate sample, there were limited numbers in some of the stakeholder groups. This was due to the study being largely located in one district. The findings for this study were mainly from interviews as opposed to observation of practice. Observational notes would have helped further with triangulation of the data and also to capture enough depth and specificity for the domain of processes.

## Conclusion and recommendations

While the lay-counselling intervention was largely reported as acceptable and helpful to providers, managers and patients, a number of system level barriers to implementation and dissemination were identified in this study. Organizational readiness emerged as one of the most important considerations that need to be addressed, with better integration of the lay-counselling service within facility operations facilitating better uptake of the service by nurse providers and patients alike. Formal recognition of counselling services provided by lay counsellors, whether designated or dedicated, was identified as potentially assisting in organizational integration of lay counsellors within the system; as was formal recognition of lay counselling as a treatment modality within mental health treatment data element definitions. The need for diversification of the roles of psychologists, especially those in internship and community service positions to include training and supervision of lay counsellors was also emphasized.

## Data Availability

The anonymised data will be made publicly available, in accordance with PRIME publication and data management policies available at https://bit.ly/2tXQQsV.

## Abbreviations

LIMCs: Low- and middle-income countries
NCDs: Non-communicable diseases
SA: South Africa
CMDs: Common mental disorders
PRIME-SA: PRogramme for Improving Mental health CarE South Africa
ICSM: Integrated clinical services model
AUD: Alcohol use disorders
APC: Adult primary care
CFIR: Consolidated framework for implementation science
HCT: HIV counselling and testing
BREC: Biomedical Research Ethics Committee
PN: Professional nurse
FM: Facility manager
DM: District manager
EAP: Employee assistance programme
CS: Counsellor supervisor
HIV: Human immunodefciency virus
AIDS: Acquired immune defciency syndrome
ART: Antiretroviral treatment
